# Relationship Between Lower Urinary Tract Problems and Quality of Life in Women with Type 2 Diabetes

**DOI:** 10.3390/healthcare14213753

**Published:** 2026-11-04

**Authors:** Demet Çelik, Ahmet Şanli, Yasemin Şanli

**Affiliations:** 1 Faculty of Health Sciences, Karamanoğlu Mehmetbey University, Karaman 70200, Türkiye; yaseminsanli@kmu.edu.tr; 2 Faculty of Medicine, Karamanoğlu Mehmetbey University, Karaman 70200, Türkiye; drasanli07@gmail.com

**Keywords:** diabetes, urinary system, quality of life

## Abstract

**Background:** This study aimed to examine the relationship between lower urinary tract symptoms and quality of life in women with type 2 diabetes. **Materials and Methods:** The population of this descriptive cross-sectional study consisted of women with type 2 diabetes who presented to the Urology, and Endocrinology and Metabolism Diseases outpatient clinics for examination between 15 May and 15 November 2025. In the study sample, 372 women were included. Data were collected through face-to-face interviews in outpatient clinics. Hierarchical multiple linear regression and sensitivity analyses were performed. **Results**: The mean age of the 372 participants was 53.23 ± 13.13 years, and the mean duration of diabetes was 8.81 years. Overall, 52.2% reported a urinary tract infection within the previous six months. The mean BFLUTS-SF total score was 28.63 ± 16.75. Higher BFLUTS-SF scores were negatively correlated with SF-36 dimensions, particularly social functioning (r = −0.562) and bodily pain (r = −0.559). Hierarchical regression analyses revealed significant negative associations between lower urinary tract symptom burden and quality-of-life dimensions after adjustment for relevant sociodemographic and clinical variables. Sensitivity analyses using the combined BFLUTS-LUTS symptom score supported these findings. **Conclusions and Recommendations**: Greater lower urinary tract symptom burden was associated with poorer health-related quality of life in women with type 2 diabetes, particularly in social functioning, bodily pain, and physical functioning. These findings highlight the importance of routinely assessing lower urinary tract symptoms alongside glycemic parameters in diabetes management. Early identification and multidisciplinary interventions may help address symptom burden and improve quality of life.

## 1. Introduction

Diabetes is a major and growing public health concern worldwide. Global estimates indicate that approximately 589 million adults aged 20–79 years were living with diabetes in 2024, representing 11.11% of this age group [1]. By 2050, this number is projected to reach 853 million, with a prevalence of 12.96%. Among women, diabetes prevalence was estimated at 10.68% in 2024 and is projected to increase to 12.73% by 2050 [1].

This burden of diabetes among women highlights the importance of assessing not only disease prevalence but also complications affecting different organs and systems [2]. Type 2 diabetes is a chronic disease that requires long-term monitoring and care and may affect daily functioning and health-related quality of life [3]. However, lower urinary tract symptoms in women with diabetes may be overlooked because they are attributed to polyuria associated with inadequate glycemic control, potentially delaying the recognition of bladder complications [4]. Reports that more than half of individuals with diabetes experience bladder dysfunction, based on data encompassing both women and men, underscore the importance of urinary symptoms in diabetes care [5]. Among women, diabetes has been associated particularly with urgency urinary incontinence and, to a lesser extent, stress urinary incontinence, with the latter association reported especially among women receiving insulin therapy [6,7].

Changes in bladder function associated with autonomic neuropathy [8], together with alterations in the detrusor muscle and urothelium, may contribute to the development of lower urinary tract symptoms in women with diabetes [9,10,11,12]. Symptoms associated with these changes are not restricted to a single clinical pattern but may encompass various problems involving urine storage and bladder emptying. In individuals with diabetes, lower urinary tract symptoms may include urinary incontinence, nocturia, increased urinary frequency, urgency, and a weak urinary stream [13]. Given that different symptoms may coexist, a comprehensive assessment of lower urinary tract symptoms in women with diabetes is important, rather than focusing solely on the presence of urinary incontinence [13].

However, these symptoms may be overlooked by healthcare professionals in women with diabetes, particularly in the early stages [14]. Attributing complaints such as urgency and increased urinary frequency solely to polyuria associated with inadequate glycemic control may delay the recognition and treatment of diabetes-related bladder complications [4]. This highlights the need to specifically inquire about and assess urinary symptoms during routine diabetes follow-up in women with type 2 diabetes [14]. The substantial burden that diabetes-related bladder dysfunction may also place on quality of life suggests that this assessment should extend beyond symptom presence and severity [3]. Therefore, diabetes care should address urinary symptoms, individual functioning, and health-related quality of life alongside glycemic control [3,14].

Assessing health-related quality of life provides insight beyond the frequency and severity of urinary symptoms into their associations with physical functioning, daily roles, social participation, and psychological well-being among women with type 2 diabetes. Previous research has shown that lower urinary tract symptoms are negatively associated with the physical and mental components of health-related quality of life in women with type 2 diabetes [3]. In a study involving both women and men with type 2 diabetes, the negative associations between urinary incontinence severity and both components remained statistically significant after adjustment for sociodemographic and clinical variables and sleep quality [15]. In Türkiye, women with type 2 diabetes were reported to experience greater incontinence-related impairment in quality of life than women with normal blood glucose levels [16]. Studies involving samples not restricted to individuals with diabetes have also demonstrated that the coexistence of multiple urinary symptoms is associated with lower physical and mental quality-of-life scores [17,18]. Incontinence-specific assessments in women have further shown that greater urinary leakage is accompanied by behavioral restrictions, social isolation, and difficulties performing religious practices [19]. However, some studies have reported significant associations of urinary incontinence or nocturia with the physical component, whereas associations with the mental component were not statistically significant, suggesting that these relationships may vary according to the symptoms and quality-of-life dimensions assessed [20,21].

The potential combined effects of female-specific pelvic floor and menopause-related changes and diabetes-related impairments in bladder sensation and function highlight the importance of examining the relationship between urinary symptoms and quality of life in women with type 2 diabetes [14,22]. Some previous studies have focused exclusively on specific complaints, such as urinary incontinence or nocturia [15,16,19,20,21], whereas others have assessed quality of life using physical and mental component summary scores [3,15,20,21]. Two of these studies focused on the relationship between lower urinary tract symptoms or urinary incontinence and quality of life in women with type 2 diabetes [3,16]. The remaining studies involved samples comprising both women and men with type 2 diabetes [15], women whose inclusion was not restricted by diabetes status [19,20], or community and primary care samples comprising women and men whose inclusion was not restricted by diabetes status [17,18,21,23]. Differences in study populations and the urinary complaints investigated limit the generalizability of these findings to women with type 2 diabetes. Collectively, these findings highlight an important research gap concerning the relationship between lower urinary tract symptoms and quality of life in this population. Accordingly, the present study aimed to examine the relationship between lower urinary tract symptoms and quality of life in women with type 2 diabetes. The findings are expected to help identify the quality-of-life domains associated with urinary symptom burden and support a more comprehensive assessment of care needs in this population. This information may guide nurses and other healthcare professionals in planning individualized care and counseling that jointly address urinary symptoms and physical, psychological, and social needs [24].

## 2. Materials and Methods

### 2.1. Type of the Study

The study is a descriptive cross-sectional study.

### 2.2. Population and Sample of the Study

Women with Type 2 diabetes who presented to the Urology, and Endocrinology and Metabolism Diseases outpatient clinics of a hospital in the Central Anatolia region of Turkey) between 15 May 2025 and 15 November 2025 and who met the inclusion criteria comprised the population of the study. The minimum required sample size was calculated using G*Power version 3.1.9.7 (Heinrich Heine University, Düsseldorf, Germany) [25], based on the correlation between lower urinary tract symptoms and the mental component summary of health-related quality of life reported by Su et al. (2023) (r = −0.26) [3]. Using a two-tailed test, a significance level of 0.05, a statistical power of 95%, and an expected correlation magnitude of 0.26, the minimum required sample size was calculated as 186 participants. To enhance the generalizability of the findings, a larger sample was recruited, and 372 women with type 2 diabetes were included using convenience sampling. A post hoc power analysis, based on the observed correlation between the BFLUTS-SF total score and the SF-36 mental health dimension (ρ = −0.253), a significance level of 0.05, and a sample size of 372, yielded an estimated statistical power of 99.87%. Convenience sampling was used for sample selection.

### 2.3. Inclusion Criteria

The inclusion criteria included being a woman 18 years of age or older, volunteering to participate in the study, having been diagnosed with Type 2 diabetes for at least 6 months, and presenting to the outpatient clinics between the specified dates.

### 2.4. Data Collection Tools

The ‘Descriptive Information Form’, ‘Bristol Female Lower Urinary Tract Symptoms Scored Form (BFLUTS-SF)’, and ‘Short Form-36 Quality of Life Scale (SF-36)’ were used as data collection tools.

***Descriptive Information Form***: The form includes items questioning the participants’ characteristics such as age, marital status, education level and their medical conditions such as duration of diabetes.

**Bristol Female Lower Urinary Tract Symptoms Scored Form (BFLUTS-SF):** The original Bristol Female Lower Urinary Tract Symptoms Questionnaire was developed by Jackson et al. (1996) [26], and its shortened, scored version was subsequently described by Brookes et al. (2004) [27]. The Turkish validity and reliability study was conducted by Güngör and Yalçın (2005) [28].

The BFLUTS-SF consists of 19 items grouped into five subscales: storage symptoms (items 1–4), voiding symptoms (items 5–7), incontinence symptoms (items 8–12), sexual function (items 13–14), and quality of life (items 15–19). Items 4, 13, 14, 17, and 19 are scored on a four-point Likert scale ranging from 0 to 3, whereas the remaining 14 items are scored on a five-point Likert scale ranging from 0 to 4. The total score ranges from 0 to 71, with higher scores indicating greater lower urinary tract symptom burden and more adverse effects on sexual function and quality of life.

The scale does not have an established cutoff score for classifying symptom severity. In the Turkish validity and reliability study, Cronbach’s alpha coefficient for the overall scale was reported as 0.66 [28]. In the present study, Cronbach’s alpha coefficient was 0.932, indicating excellent internal consistency in the study sample.

***Short Form-36 Quality of Life Scale (SF-36)***

The SF-36 was originally developed by Ware and Sherbourne (1992) [29], and its Turkish validity and reliability were established by Koçyiğit et al. (1999) [30]. It is a generic, self-administered instrument designed to assess health-related quality of life across physical, psychological, and social domains, regardless of specific diseases or age groups.

The SF-36 comprises 36 items organized into eight dimensions: physical functioning (10 items), role physical (4 items), role emotional (3 items), vitality (4 items), mental health (5 items), social functioning (2 items), bodily pain (2 items), and general health (5 items). An additional item evaluates perceived changes in health over the preceding year and is not included in the eight dimension scores. The remaining items assess current health status and health experiences over varying reference periods, including the preceding four weeks. Each dimension is scored separately on a scale ranging from 0 to 100, with higher scores indicating better health-related quality of life.

In the original Turkish validation study, Cronbach’s alpha coefficients were 0.7529 for physical functioning, 0.7576 for role physical, 0.7612 for role emotional, 0.7324 for vitality, 0.7610 for mental health, 0.7529 for social functioning, 0.7602 for bodily pain, and 0.7593 for general health [30]. In the present study (*n* = 372), the corresponding Cronbach’s alpha coefficients were 0.984, 0.893, 0.833, 0.665, 0.703, 0.594, 0.919, and 0.802, respectively.

### 2.5. Data Collection

Data were collected by the researchers through face-to-face interviews in the urology and endocrinology and metabolism outpatient clinics between 09:00 a.m. and 12:00 noon. A history of urinary tract infection within the previous six months was ascertained by reviewing the hospital’s electronic medical records, including physician-documented diagnoses, accompanying urinary symptoms, urinalysis and urine culture results, and the dates and number of infection episodes. These records were reviewed to assess symptomatic status, laboratory confirmation, and recurrence. For the analyses, urinary tract infection history was coded as a binary variable indicating the presence or absence of a documented infection during the preceding six months.

### 2.6. Data Analysis

Data were analyzed using the IBM SPSS (International Business Machines Corporation, Statistical Package for the Social Sciences) version 21.0 (Armonk, NY, USA: IBM Corp). Descriptive statistics were used to summarize participants’ sociodemographic and clinical characteristics, lower urinary tract symptom scores, and health-related quality-of-life scores. Categorical variables were presented as frequencies and percentages, whereas continuous variables were summarized as means and standard deviations.

The distributional characteristics of the overall BFLUTS-SF score, the BFLUTS-LUTS combined symptom score, and the eight SF-36 dimension scores were evaluated using the Kolmogorov–Smirnov test with Lilliefors correction, the Shapiro–Wilk test, skewness and kurtosis coefficients, histograms, and normal Q–Q plots. Both normality tests indicated statistically significant deviations from normality across all scale scores (all *p* < 0.001). Graphical assessments also revealed departures from normality, particularly for the physical functioning, role physical, and role emotional dimensions of the SF-36. Detailed results of these assessments are provided in Table 1.

Given these distributional characteristics, Spearman’s rank correlation analysis was used to examine the associations between lower urinary tract symptom burden and health-related quality of life. Spearman’s rank correlation coefficients (r) were calculated for the relationships between each of the two urinary symptom scores (overall BFLUTS-SF and BFLUTS-LUTS) and the eight SF-36 dimensions, yielding 16 correlation analyses. The BFLUTS-LUTS combined symptom score was designated as the primary urinary symptom measure for the correlation analyses, while the overall BFLUTS-SF score was examined in secondary analyses. Spearman’s ρ was interpreted as an effect-size measure, and 95% confidence intervals were estimated using nonparametric bootstrap resampling with 5000 samples. All statistical tests were two-tailed, with a nominal significance level of *p* < 0.05. To account for multiple comparisons across the 16 correlation analyses, the Bonferroni correction was applied, resulting in an adjusted significance threshold of *p* < 0.003125.

Hierarchical multiple linear regression analyses were conducted separately for each of the eight SF-36 dimensions to examine their associations with the overall BFLUTS-SF score after adjustment for potential confounding variables. Sociodemographic and clinical covariates were entered simultaneously in the first block, including age, menopausal status, diabetes duration, body mass index, glycated hemoglobin (HbA1c), history of urinary tract infection, and comorbidity status. The overall BFLUTS-SF score was subsequently entered in the second block to evaluate its additional contribution beyond these covariates. In addition to the five BFLUTS-SF subscale scores and the overall BFLUTS-SF score, a combined lower urinary tract symptom score (BFLUTS-LUTS) was calculated by summing the storage (0–15), voiding (0–12), and incontinence (0–20) subscale scores, based on the subscale scoring approach described by Brookes et al. [27]. As a sensitivity analysis, the regression models were repeated by replacing the overall BFLUTS-SF score with the BFLUTS-LUTS combined symptom score. This analysis was intended to determine whether the associations persisted when the sexual function and quality-of-life components of the BFLUTS-SF were excluded, thereby reducing potential conceptual overlap with the SF-36 outcomes.

The assumptions of hierarchical multiple linear regression were evaluated separately for each SF-36 subscale model. Residual independence was examined using the Durbin–Watson statistic, with values ranging from 1.782 to 2.178, indicating no substantial evidence of first-order autocorrelation. Multicollinearity was assessed using variance inflation factors and tolerance values, which did not indicate substantial multicollinearity. Residual normality was evaluated through histograms and normal probability (P-P) plots, whereas linearity was examined using partial regression plots and standardized residual-versus-predicted-value plots. Visual inspection suggested approximately normal residual distributions in the models. No consistent curvilinear patterns or pronounced funnel-shaped dispersion was evident.

Homoscedasticity was further assessed using the Breusch–Pagan test, which indicated evidence of heteroscedasticity in three of the eight regression models: role physical (χ^2^(8) = 26.825, *p* < 0.001), role emotional (χ^2^(8) = 23.677, *p* = 0.003), and mental health (χ^2^(8) = 20.408, *p* = 0.009). Accordingly, heteroscedasticity-consistent HC3 standard errors were calculated for all eight models to evaluate the robustness of the statistical inferences. The negative associations between the overall BFLUTS-SF score and all eight SF-36 dimensions remained statistically significant after HC3 adjustment (all *p* < 0.05).

Potentially influential observations were evaluated using Cook’s distance, leverage values, Mahalanobis distance, studentized residuals, DFFITS, and DFBETAs. Maximum Cook’s distance values across the eight models ranged from 0.046 to 0.373. Although none exceeded the conventional reference value of 1, some observations exhibited elevated influence statistics, indicating that potentially influential cases could not be excluded solely on the basis of Cook’s distance.

### 2.7. Ethical Issues

Before the study was conducted, ethical approval was obtained from the Karamanoğlu Mehmetbey University Health Sciences Scientific Research Ethics Committee (03-2025/29) and institutional permission from the management of the hospital (date: 30 April 2025, number: 20). Written consent was obtained from the participants, after they were informed about the scope of the study. All procedures involving human participants were conducted in accordance with the ethical principles of the World Medical Association Declaration of Helsinki, originally adopted in 1964 and most recently revised in October 2024.

## 3. Results

The mean age of the participants was 53.23 ± 13.13 years. Of the participants, most were unemployed, 62.1% were in menopause, 37.9% had another chronic disease comorbid with diabetes, and 52.2% had a urinary tract infection in the last six months. The mean duration of having diabetes was 8.81 years (Table 2).

The mean overall BFLUTS-SF score was 28.63 ± 16.75, while the mean BFLUTS-LUTS combined symptom score was 18.66 ± 11.00. The mean scores for the SF-36 dimensions were as follows: 59.69 ± 36.39 for physical functioning, 55.75 ± 23.55 for social functioning, 47.98 ± 43.51 for role physical, 49.46 ± 43.33 for role emotional, 55.41 ± 15.39 for mental health, 50.35 ± 16.48 for vitality, 57.86 ± 31.20 for bodily pain, and 49.29 ± 18.82 for general health (Table 3).

Spearman’s rank correlation analysis revealed negative associations between the BFLUTS-SF total score and all eight SF-36 dimensions (Table 4). The strongest correlations were observed for social functioning (ρ = −0.562, 95% CI [−0.636, −0.481], *p* < 0.001) and bodily pain (ρ = −0.559, 95% CI [−0.641, −0.475], *p* < 0.001), followed by physical functioning (ρ = −0.427, 95% CI [−0.514, −0.331], *p* < 0.001), role physical (ρ = −0.310, 95% CI [−0.402, −0.215], *p* < 0.001), role emotional (ρ = −0.279, 95% CI [−0.371, −0.183], *p* < 0.001), mental health (ρ = −0.253, 95% CI [−0.351, −0.148], *p* < 0.001), general health (ρ = −0.243, 95% CI [−0.345, −0.142], *p* < 0.001), and vitality (ρ = −0.108, 95% CI [−0.213, −0.003], *p* = 0.037) (Table 4).

Similarly, the BFLUTS-LUTS combined symptom score was negatively correlated with social functioning (ρ = −0.560, 95% CI [−0.634, −0.478], *p* < 0.001), bodily pain (ρ = −0.559, 95% CI [−0.638, −0.477], *p* < 0.001), physical functioning (ρ = −0.412, 95% CI [−0.500, −0.316], *p* < 0.001), role physical (ρ = −0.294, 95% CI [−0.387, −0.201], *p* < 0.001), role emotional (ρ = −0.255, 95% CI [−0.348, −0.160], *p* < 0.001), mental health (ρ = −0.246, 95% CI [−0.345, −0.143], *p* < 0.001), general health (ρ = −0.244, 95% CI [−0.345, −0.143], *p* < 0.001), and vitality (ρ = −0.117, 95% CI [−0.219, −0.012], *p* = 0.024) (Table 4).

After applying the Bonferroni correction for 16 comparisons (adjusted significance threshold: *p* < 0.003125), 14 of the 16 associations remained statistically significant. The correlations between vitality and the BFLUTS-SF total score (*p* = 0.037) and BFLUTS-LUTS combined symptom score (*p* = 0.024) did not remain statistically significant after correction. Overall, greater lower urinary tract symptom burden was associated with poorer health-related quality of life, particularly in the social functioning and bodily pain dimensions (Table 4).

After adjustment for age, menopausal status, diabetes duration, body mass index, HbA1c, history of urinary tract infection, and comorbidity status, adding the BFLUTS-SF total score significantly increased the explained variance in the SF-36 dimensions (ΔR^2^ = 0.058–0.308, *p* ≤ 0.004). Higher BFLUTS-SF scores were associated with lower scores across the SF-36 dimensions (standardized β = −0.289 to −0.667). The strongest associations were observed for social functioning (β = −0.667; adjusted R^2^ = 0.486) and bodily pain (β = −0.639; adjusted R^2^ = 0.528). Although the BFLUTS-SF coefficient for general health was significant (β = −0.289, *p* = 0.004), the overall final model was not statistically significant (*p* = 0.059); therefore, this finding should be interpreted cautiously. Detailed results are presented in Supplementary Table S1.

To minimize potential conceptual overlap between the BFLUTS-SF quality-of-life items and the SF-36 domains, sensitivity analyses were conducted using the BFLUTS-LUTS combined storage, voiding, and incontinence symptom score. After adjustment for age, menopausal status, diabetes duration, body mass index, HbA1c, history of urinary tract infection, and chronic disease status, higher BFLUTS-LUTS scores were negatively associated with all eight SF-36 dimensions. Adding the BFLUTS-LUTS score significantly increased the explained variance across dimensions (ΔR^2^ = 0.059–0.314, all *p* ≤ 0.004).

The strongest associations were observed for social functioning (β = −0.676, *p* < 0.001; adjusted R^2^ = 0.493) and bodily pain (β = −0.636, *p* < 0.001; adjusted R^2^ = 0.523). Negative associations were also found for physical functioning (β = −0.495; adjusted R^2^ = 0.356), vitality (β = −0.489; adjusted R^2^ = 0.266), mental health (β = −0.453; adjusted R^2^ = 0.251), role physical (β = −0.449; adjusted R^2^ = 0.233), and role emotional (β = −0.398; adjusted R^2^ = 0.222), with all coefficients significant at *p* < 0.001.

Among the covariates retained in the final models, longer diabetes duration was negatively associated with vitality (β = −0.242, *p* = 0.004) and mental health (β = −0.331, *p* < 0.001). Older age was positively associated with bodily pain scores (β = 0.251, *p* = 0.014), whereas higher HbA1c was negatively associated with these scores (β = −0.150, *p* = 0.032).

For general health, the BFLUTS-LUTS coefficient was significant (β = −0.292, *p* = 0.004), but the overall final model did not reach statistical significance (*p* = 0.057; adjusted R^2^ = 0.052). Overall, the sensitivity analyses yielded findings consistent with the primary analyses, with comparable standardized coefficients and adjusted R^2^ values. Detailed results are presented in Supplementary Table S2.

## 4. Discussion

The present study demonstrated a consistent negative association between lower urinary tract symptom burden and health-related quality of life among women with type 2 diabetes. Hierarchical multiple linear regression analyses showed that higher BFLUTS-SF total scores were associated with poorer outcomes across all eight SF-36 dimensions after adjustment for relevant sociodemographic and clinical factors. Importantly, sensitivity analyses using the BFLUTS-LUTS combined symptom score yielded comparable standardized regression coefficients (β = −0.667 to −0.289 in the primary models and β = −0.676 to −0.292 in the sensitivity models) and adjusted R^2^ values (0.051–0.528 and 0.052–0.523, respectively). The strongest associations were consistently observed for social functioning and bodily pain. These findings suggest that the relationship between urinary symptom burden and health-related quality of life was not solely attributable to the conceptual overlap arising from the sexual function and quality-of-life components of the overall BFLUTS-SF score.

Beyond social functioning and bodily pain, higher urinary symptom burden was associated with poorer physical functioning, greater role limitations due to physical and emotional problems, and lower mental health scores. The findings for vitality were less consistent across analytical approaches. Its weak bivariate correlations with both urinary symptom measures did not remain statistically significant after Bonferroni correction, although the associations were significant in the adjusted regression models. General health showed the smallest standardized regression coefficients and the lowest adjusted R^2^ values. Although the urinary symptom coefficients were statistically significant in both analyses, the overall general health models did not reach statistical significance, warranting cautious interpretation. Together, these findings indicate that the strength and consistency of the associations varied across health-related quality-of-life dimensions.

Consistent with the findings of the present study, Su et al. (2023) [3], in a study conducted among women with type 2 diabetes in Taiwan, reported that lower urinary tract symptoms were negatively associated with the mental and physical component summary scores of health-related quality of life. Similar associations were demonstrated in a cross-sectional study conducted by Chawla et al. (2026) [15] among 300 women and men with type 2 diabetes in Pakistan. In that study, in which 60% of participants were women, urinary incontinence severity was negatively associated with both physical and mental component summary scores, and these associations remained statistically significant after adjustment for sociodemographic and clinical variables and sleep quality [15]. These findings support an association between urinary symptoms and both the physical and mental aspects of quality of life in individuals with type 2 diabetes.

Findings from Türkiye concerning women with type 2 diabetes are also consistent with these results. In a study comparing 143 women with type 2 diabetes and 147 women with normal blood glucose levels, Ergenoğlu et al. (2012) [16] reported that severe urgency and stress urinary incontinence complaints were more common among women with type 2 diabetes. Furthermore, scores on the scale assessing the adverse impact of incontinence on quality of life were significantly higher among women with type 2 diabetes than in the comparison group [16].

Studies involving samples not restricted to individuals with diabetes also provide evidence of an association between urinary symptom burden and quality of life. In a community-based study conducted in the United States among 3201 women and 2301 men aged 30–79 years, Hall et al. (2009) [17] reported lower physical and mental component summary scores in the groups with the greatest number of coexisting urological symptoms. In analyses adjusted for age, race/ethnicity, and comorbidities, women in the most symptomatic group had physical and mental component summary scores that were 9.80 and 6.24 points lower, respectively, than those of asymptomatic women [17]. Both differences were statistically significant, supporting an association between the coexistence of multiple urinary symptoms and both the physical and mental aspects of quality of life in women.

Similar findings regarding coexisting symptoms were reported by Choi et al. (2020) [18] in a cross-sectional study conducted in Hong Kong among 287 female and 213 male primary care patients aged 40 years and older, whose inclusion was not restricted to individuals with type 2 diabetes. Participants with lower urinary tract symptoms had lower physical and mental component summary scores. In analyses adjusted for sociodemographic factors, participants with coexisting storage, voiding, and post-micturition symptoms had lower scores across all eight quality-of-life dimensions. Their physical and mental component summary scores were 6.3 and 6.7 points lower, respectively, than those of participants without symptoms. However, participants with storage symptoms alone did not differ significantly from those without symptoms in either physical or mental component summary scores [18]. These findings suggest that associations with quality of life may vary according to the type and coexistence of urinary symptoms.

The relationship between urinary symptom severity and health status has also been examined in earlier community-based research. Although published some time ago, a study conducted by Boyle et al. (2003) [23] among 4800 men and 3674 women in the Netherlands, France, the United Kingdom, and Korea reported that physical and mental component scores decreased with increasing lower urinary tract symptom severity in both women and men. Women with the same urinary symptom scores as men also reported poorer health status [23].

However, associations with the physical and mental components have not been equally consistent across studies. In an 11-year longitudinal study involving women whose inclusion was not restricted to those with type 2 diabetes, Avis et al. (2018) [20] found that women experiencing urinary incontinence at least once a week had lower physical component summary scores than women who never experienced urinary incontinence. In contrast, the negative association between urinary incontinence and mental component summary scores observed in bivariate analyses was no longer statistically significant in the final multivariable model. Similarly, in a study conducted in Hong Kong among female and male primary care patients aged 40 years and older, whose inclusion was not restricted to individuals with type 2 diabetes, Choi et al. (2019) [21] found that participants experiencing nocturia at least twice per night had lower physical component summary scores, whereas no statistically significant difference was observed in mental component summary scores. Despite differences in sample characteristics, these findings support the negative associations between urinary symptom burden and the physical dimensions of quality of life identified in the present study, while indicating that findings concerning the mental dimensions may vary across studies.

In addition to studies assessing general health-related quality of life, research focusing on incontinence-specific quality of life highlights the difficulties women experience in their daily and social lives. In a cross-sectional study conducted in Türkiye among 65 women presenting to a university hospital with urinary incontinence, whose inclusion was not restricted to individuals with type 2 diabetes, Rüzgar et al. (2020) [19] reported that greater urinary leakage, objectively measured using a pad test, was associated with poorer quality of life and greater disruption to the spiritual dimension of women’s lives. Women with more than 10 g of urinary leakage during a one-hour pad test had lower total and subscale scores for incontinence-specific quality of life, restricted their activities more, experienced greater social isolation, and had more difficulty performing religious practices. However, quality-of-life scores did not differ significantly by urinary incontinence type [19].

Differences between the present study and previous research may be related to sample characteristics, the range of urinary symptoms assessed, the measurement of quality of life, and the variables considered in the analyses. Whereas the present study included only women with type 2 diabetes, some studies involved both women and men with type 2 diabetes or samples not restricted to individuals with diabetes [15,17,18,21]. Differences in age distribution, menopausal status, diabetes duration, comorbidities, and symptom severity may also have influenced the magnitude of the associations. Indeed, the studies compared included diverse samples, ranging from women followed from midlife into older age to primary care patients aged 40 years and older and adults with type 2 diabetes (Avis et al., 2018; Choi et al., 2019, 2020; Chawla et al., 2026) [15,18,20,21]. Furthermore, studies assessing nocturia or urinary incontinence alone do not capture the same symptom burden as the present study, which examined storage, voiding, and incontinence symptoms together [15,20,21]. The physical and mental component summary scores used in previous studies are also not directly equivalent to the eight quality-of-life dimensions assessed separately in the present study [15,17,20,21]. Therefore, differences in findings, particularly those concerning mental health, may reflect variations in the constructs measured and statistical adjustments, as well as sample characteristics. For example, the association between urinary incontinence and mental component summary scores was no longer statistically significant in the final multivariable model in one study, whereas it remained significant after adjustment for sleep quality and other variables in another [15,20].

The strongest associations observed in the present study for social functioning and bodily pain may reflect the implications of urinary symptoms for different aspects of daily life. Urinary frequency, urgency, and the possibility of urinary leakage may lead women to organize their social activities around toilet availability and limit the time they spend outside the home. Concerns about noticeable leakage or odor may also contribute to avoidance of social situations. However, because these behaviors were not directly assessed in the present study, they should be considered potential explanations for the association with social functioning. The negative association with bodily pain scores suggests that greater urinary symptom burden may coexist with greater pain and pain-related interference with daily life. However, this dimension does not measure urinary tract-specific pain, so the association should not be attributed solely to urinary complaints. Neuropathic or musculoskeletal pain associated with diabetes, along with other unmeasured health conditions, may also have contributed to this finding.

The associations observed with physical functioning and role limitations due to physical health problems may be explained by the potentially restrictive effects of urinary symptoms on mobility and the ability to fulfill daily responsibilities. In particular, concerns about urinary leakage during movement or the need for frequent toilet visits may hinder participation in walking, exercise, household tasks, and other daily activities. Urinary complaints occurring alongside existing physical difficulties associated with type 2 diabetes may further complicate these activities. The unpredictability of symptoms, a sense of loss of control, embarrassment, and the need to continually plan daily life around symptoms may contribute to the associations observed with mental health and role limitations due to emotional problems. However, because the cross-sectional design does not allow the direction of these relationships to be determined, poorer physical and mental health may also be associated with symptom perception and reporting.

The variation in vitality findings across analyses also warrants consideration. The weak bivariate correlations, which did not remain statistically significant after adjustment for multiple comparisons, suggest a limited unadjusted association between urinary symptom burden and energy and fatigue levels. In contrast, the significant associations observed in the adjusted regression models may reflect a different pattern of association after accounting for covariates. Differences in the statistical properties of correlation and regression analyses, together with the application of multiple-comparison correction to the correlation analyses, may also have contributed to the differing significance results. Therefore, this finding should not be interpreted as definitive evidence that the association became stronger after adjustment. Although nocturia-related sleep fragmentation and the burden of coping with symptoms are potential explanations for lower vitality, sleep quality was not assessed in the present analyses, precluding direct examination of this explanation.

The smaller standardized coefficients and lower adjusted R^2^ values observed for general health may reflect the influence of multiple factors beyond urinary symptoms on overall health perceptions. The course of diabetes, other health conditions, treatment burden, and expectations regarding future health may contribute to women’s assessments in this dimension. Although the urinary symptom coefficient was statistically significant, the overall model did not reach statistical significance, warranting more cautious interpretation of these findings. Furthermore, the similar results obtained using the combined symptom score excluding the sexual function and quality-of-life components support the interpretation that the observed associations cannot be explained solely by conceptual overlap between the instruments. Nevertheless, this consistency does not rule out potential contributions from unmeasured confounders, the shared self-report method, or reverse associations.

The strengths of the present study include the assessment of lower urinary tract symptoms and health-related quality of life in 372 women with type 2 diabetes using instruments with established validity and reliability, the separate examination of eight quality-of-life dimensions, and the application of hierarchical regression analyses adjusted for relevant sociodemographic and clinical variables. The similar results obtained in sensitivity analyses using a combined symptom score excluding the sexual function and quality-of-life components support the consistency of the findings. Furthermore, the use of Bonferroni correction for multiple correlation analyses and heteroscedasticity-consistent standard errors in the regression analyses strengthens the statistical assessment. Nevertheless, the study has several limitations. Its cross-sectional design precludes determining the temporal direction of the associations or establishing causality. The absence of a non-diabetic comparison group prevents the observed urinary symptom burden and differences in quality of life from being attributed directly to type 2 diabetes. Recruiting women attending urology and endocrinology outpatient clinics at a single center through convenience sampling may introduce selection bias and limit the generalizability of the findings to all women with type 2 diabetes in the community or to other healthcare settings. The reliance on self-reported urinary symptom and quality-of-life data may introduce recall, social desirability, and common method biases. Despite adjustment for relevant sociodemographic and clinical variables, residual confounding from unmeasured factors, including sleep quality, depressive symptoms, diabetic neuropathy, obstetric history, and medication use, cannot be excluded. Although Bonferroni correction was applied to the correlation analyses, the evaluation of multiple regression models and coefficients may increase the likelihood of false-positive findings. In addition, the relatively low internal consistency of the social functioning dimension, elevated influence statistics for some observations, and the lack of overall statistical significance in the general health models warrant cautious interpretation of the relevant findings.

## 5. Conclusions and Recommendations

The present study found that lower urinary tract symptom burden was negatively associated with all eight dimensions of quality of life among women with type 2 diabetes after adjustment for relevant sociodemographic and clinical variables, with the strongest associations observed for social functioning and bodily pain. Similar results in the sensitivity analyses support the interpretation that these associations cannot be explained solely by conceptual overlap between the instruments. However, differences in vitality findings across analyses and the lack of overall statistical significance in the general health models warrant cautious interpretation of these dimensions. Given the negative associations between urinary symptom burden and all eight quality-of-life dimensions, care for women with type 2 diabetes should include an assessment of physical, psychological, and social needs alongside urinary complaints. Nurses should pay particular attention to social functioning and bodily pain, exploring barriers to social participation and the impact of pain on daily life, and planning individualized care based on identified needs. Difficulties maintaining physical activities and daily roles, emotional distress, energy levels, and general health perceptions should also be included in nursing assessments. Regular monitoring of quality of life and referral to appropriate specialists according to individual needs are recommended for women with a high urinary symptom burden.

## Figures and Tables

**Table 1:** Normality assessment of BFLUTS-SF and SF-36 scores.

Variable	Skewness	Kurtosis	K–S D	K–S *p*	S–W W	S–W *p*
**BFLUTS-SF scores**						
BFLUTS-SF total score	0.170	−0.882	0.073	<0.001	0.970	<0.001
BFLUTS-LUTS combined symptom score	0.339	−0.675	0.089	<0.001	0.966	<0.001
**SF-36 dimensions**						
Physical functioning	−0.360	−1.148	0.189	<0.001	0.849	<0.001
Role physical	0.109	−1.760	0.223	<0.001	0.776	<0.001
Role emotional	0.047	−1.720	0.241	<0.001	0.779	<0.001
Vitality	−0.366	0.178	0.147	<0.001	0.970	<0.001
Mental health	−0.412	0.743	0.124	<0.001	0.973	<0.001
Social functioning	−0.188	−0.493	0.140	<0.001	0.958	<0.001
Bodily pain	−0.454	−0.833	0.155	<0.001	0.920	<0.001
General health	−0.541	1.023	0.147	<0.001	0.938	<0.001

Note. BFLUTS-SF = Bristol Female Lower Urinary Tract Symptoms Scored Form; BFLUTS-LUTS = combined lower urinary tract symptom score; SF-36 = 36-Item Short Form Health Survey; K–S = Kolmogorov–Smirnov test (Lilliefors correction); S–W = Shapiro–Wilk test; D and W = test statistics. Standard errors: skewness = 0.126; kurtosis = 0.252. All normality tests were based on *n* = 372. SPSS outputs of 0.000 are presented as *p* < 0.001.

**Table 2:** Descriptive characteristics of the participants.

Characteristics		
Mean age: 53.23 ± 13.13 (Minimum: 20; Maximum: 65) years Mean height: 160.33 cm Mean weight: 82.34 kg Mean duration of having diabetes: 8.81 years Fasting blood glucose level: 166.15 ± 84 mg/dL Hemoglobin A1c level: %8.00 ± 2.15		
**Variables**	**Number**	**Percentage**
**Income status**		
Income less than expenses	45	12.1
Income equal to expenses	307	82.5
Income more than expenses	20	5.4
**Education**		
Primary school	191	51.3
Junior high school	44	11.8
Senior high school	54	14.5
Higher education	83	22.3
**Employment status**		
Not employed	192	51.6
Civil servant	86	23.1
Retired	51	13.7
Others	43	11.6
**Marital status**		
Single	31	8.3
Married	341	91.7
**Smoking**		
Yes	75	20.2
No	297	79.8
**Being in menopause**		
Yes	231	62.1
No	141	37.9
**Presence of a chronic disease comorbid with diabetes**		
Yes	141	37.9
No	231	62.1
**Having a urinary tract infection**		
Yes	194	52.2
No	178	47.8

**Table 3:** Mean scores the participants obtained from the overall ‘Bristol Female Lower Urinary Tract Symptoms Questionnaire (FLUTSQ)’ and its dimensions, and the dimensions of the ‘Short Form-36 Quality of Life Scale (SF-36)’.

	Minimum and Maximum Scores	Mean/Standard Deviation
**BFLUTS-SF Total Score**	0–71	28.63 ± 16.75
**BFLUTS-SF Subscales**		
Storage symptoms	0–15	7.02 ± 3.48
Voiding symptoms	0–12	4.50 ± 3.36
Incontinence symptoms	0–20	7.14 ± 5.01
Sexual function	0–6	1.64 ± 1.94
Quality of life	0–18	8.33 ± 5.44
**BFLUTS-LUTS Combined Symptom Score**	0–47	18.66 ± 11.00
**SF-36 Subscales**		
Physical functioning	0–100	59.69 ± 36.39
Role physical	0–100	47.98 ± 43.51
Role emotional	0–100	49.46 ± 43.33
Vitality	0–100	50.35 ± 16.48
Mental health	4–100	55.41 ± 15.39
Social functioning	0–100	55.75 ± 23.55
Bodily pain	0–100	57.86 ± 31.20
General health	0–100	49.29 ± 18.82

Note. BFLUTS-SF = Bristol Female Lower Urinary Tract Symptoms Scored Form; BFLUTS-LUTS = combined lower urinary tract symptom score; SF-36 = 36-Item Short Form Health Survey. The BFLUTS-LUTS combined symptom score was calculated by summing the storage (0–15), voiding (0–12), and incontinence (0–20) subscale scores of the BFLUTS-SF, yielding a total possible score ranging from 0 to 47. Minimum–maximum values represent observed scores in the study sample.

**Table 4:** Correlations of BFLUTS-SF total and BFLUTS-LUTS combined symptom scores with the SF-36 dimensions (*n* = 372).

SF-36 Dimension	BFLUTS-SF Total Score		BFLUTS-LUTS Combined Score	
	r	*p*	r	*p*
Physical functioning	−0.427	<0.001	−0.412	<0.001
Role physical	−0.310	<0.001	−0.294	<0.001
Role emotional	−0.279	<0.001	−0.255	<0.001
Vitality	−0.108	0.037	−0.117	0.024
Mental health	−0.253	<0.001	−0.246	<0.001
Social functioning	−0.562	<0.001	−0.560	<0.001
Bodily pain	−0.559	<0.001	−0.559	<0.001
General health	−0.243	<0.001	−0.244	<0.001

Note. Values are Spearman rank correlation coefficients (r) and two-sided *p* values. BFLUTS-SF = Bristol Female Lower Urinary Tract Symptoms Scored Form; BFLUTS-LUTS = combined lower urinary tract symptom score; SF-36 = 36-Item Short Form Health Survey. After Bonferroni correction for 16 comparisons (α = 0.003125), only the two vitality correlations were not statistically significant.

## Data Availability

The data supporting the conclusions of this article will be made available by the authors on request.

## Supplementary Materials

The following supporting information can be downloaded at https://www.mdpi.com/article/10.3390/healthcare14213753/s1, Table S1: Hierarchical regression analysis of the association between BFLUTS-SF total score and SF-36 sub-scales; Table S2: Sensitivity analysis of the association between the BFLUTS-LUTS score and SF-36 subscales using hierarchical multiple linear regression.
