# Supplementary material for: Relationship Between Lower Urinary Tract Problems and Quality of Life in Women with Type 2 Diabetes
Source: Healthcare (Basel). 2026 Nov 4;14(21):3753. doi: 10.3390/healthcare14213753 (PMC13762720; doi:10.3390/healthcare14213753)
Supplement: Supplementary file 1 [file healthcare-14-03753-s001.zip › healthcare-4622309-supplementary.pdf]

Supplementary Table S1. Hierarchical regression analysis of the association between BFLUTS-SF total score and SF-36 subscales.

Physical Functioning

Model 1

Variables	Unstandardized coefficient		Standardized coefficient	t	p	95% CI		Collinearity statistics	
	B	SE	Beta			Lower	Upper	Tolerance	VIF
(Constant)	105,662	28,916		3,654	,000	48,463	162,862		
Age (years)	,209	,376	,073	,555	,580	-,536	,953	,341	2,932
Menopause=No	10,239	9,253	,134	1,107	,270	-8,064	28,542	,399	2,504
Duration of diabetes (years)	-,839	,422	-,174	-1,989	,049	-1,673	-,004	,767	1,305
Body mass index (kg/m²)	-,393	,404	-,083	-,972	,333	-1,193	,407	,802	1,248
HbA1c (%)	-6,339	1,574	-,333	-4,028	,000	-9,451	-3,226	,860	1,162
History of urinary tract infection=No	4,093	6,080	,053	,673	,502	-7,934	16,120	,956	1,046
Chronic disease=No	1,307	7,259	,017	,180	,857	-13,052	15,666	,689	1,452

Note: F=5,463, $p < 0.001$; Adj. R^2=0.184. Variables included in the regression model: age, menopausal status (reference category: yes), duration of diabetes, body mass index, HbA1c, history of urinary tract infection (reference category: yes), presence of chronic disease (reference category: yes).

Model 2

Variables	Unstandardized coefficient		Standardized coefficient	t	p	95% CI		Collinearity statistics	
	B	SE	Beta			Lower	Upper	Tolerance	VIF
(Constant)	99,043	25,876		3,828	,000	47,854	150,232		
Age (years)	,377	,338	,131	1,116	,266	-,291	1,045	,339	2,954
Menopause=No	10,596	8,272	,139	1,281	,203	-5,769	26,960	,399	2,504
Duration of diabetes (years)	-,752	,377	-,156	-1,993	,048	-1,499	-,006	,765	1,307
Body mass index (kg/m²)	-,095	,365	-,020	-,261	,795	-,817	,627	,786	1,272
HbA1c (%)	-2,474	1,554	-,130	-1,592	,114	-5,549	,601	,705	1,419
History of urinary tract infection=No	,847	5,464	,011	,155	,877	-9,962	11,656	,946	1,057
Chronic disease=No	,477	6,491	,006	,073	,942	-12,364	13,318	,688	1,453

BFLUTS-SF total score	-1,224	,210	-,481	-5,845	,000	-1,639	-,810	,693	1,442

Note: F=10,250, p < 0.001; Adj. R^2=0.347; ΔR^2=0.160, F change=34,158, p < 0.001. Variables included in the regression model: age, menopausal status (reference category: yes), duration of diabetes, body mass index, HbA1c, history of urinary tract infection (reference category: yes), presence of chronic disease (reference category: yes), BFLUTS-SF total score.

Role Physical

Model 1

Variables	Unstandardized coefficient		Standardized coefficient	t	p	95% CI		Collinearity statistics	
	B	SE	Beta			Lower	Upper	Tolerance	VIF
(Constant)	62,703	34,110		1,838	,068	-4,770	130,176		
Age (years)	,320	,444	,100	,721	,472	-,558	1,198	,341	2,932
Menopause=No	10,411	10,915	,122	,954	,342	-11,179	32,001	,399	2,504
Duration of diabetes (years)	-,311	,498	-,058	-,625	,533	-1,295	,673	,767	1,305
Body mass index (kg/m²)	-,075	,477	-,014	-,158	,875	-1,019	,868	,802	1,248
HbA1c (%)	-5,878	1,856	-,276	-3,167	,002	-9,550	-2,207	,860	1,162
History of urinary tract infection=No	8,646	7,172	,100	1,205	,230	-5,541	22,833	,956	1,046
Chronic disease=No	7,653	8,563	,087	,894	,373	-9,286	24,591	,689	1,452

Note: F=3,013, p = 0.006; Adj. R^2=0.092. Variables included in the regression model: age, menopausal status (reference category: yes), duration of diabetes, body mass index, HbA1c, history of urinary tract infection (reference category: yes), presence of chronic disease (reference category: yes).

Model 2

Variables	Unstandardized coefficient		Standardized coefficient	t	p	95% CI		Collinearity statistics	
	B	SE	Beta			Lower	Upper	Tolerance	VIF
(Constant)	55,896	31,465		1,776	,078	-6,349	118,141		
Age (years)	,493	,411	,154	1,201	,232	-,319	1,305	,339	2,954
Menopause=No	10,778	10,059	,126	1,071	,286	-9,121	30,676	,399	2,504
Duration of diabetes (years)	-,222	,459	-,041	-,484	,629	-1,130	,686	,765	1,307
Body mass index (kg/m²)	,231	,444	,044	,520	,604	-,647	1,109	,786	1,272
HbA1c (%)	-1,905	1,890	-,089	-1,008	,315	-5,644	1,834	,705	1,419
History of urinary tract infection=No	5,308	6,644	,061	,799	,426	-7,836	18,451	,946	1,057
Chronic disease=No	6,799	7,893	,077	,861	,391	-8,815	22,414	,688	1,453

BFLUTS-SF total score	-1,259	,255	-,442	-4,942	,000	-1,763	-,755	,693	1,442

Note: F=6,157, $p < 0.001$; Adj. R^2=0.229; ΔR^2=0.135, F change=24,424, $p < 0.001$. Variables included in the regression model: age, menopausal status (reference category: yes), duration of diabetes, body mass index, HbA1c, history of urinary tract infection (reference category: yes), presence of chronic disease (reference category: yes), BFLUTS-SF total score.

Role Emotional

Model 1

Variables	Unstandardized coefficient		Standardized coefficient	t	p	95% CI		Collinearity statistics	
	B	SE	Beta			Lower	Upper	Tolerance	VIF
(Constant)	75,359	33,842		2,227	,028	8,417	142,302		
Age (years)	,288	,440	,089	,653	,515	-,583	1,159	,341	2,932
Menopause=No	8,472	10,829	,099	,782	,435	-12,948	29,893	,399	2,504
Duration of diabetes (years)	-,468	,494	-,087	-,948	,345	-1,445	,508	,767	1,305
Body mass index (kg/m²)	-,159	,473	-,030	-,335	,738	-1,095	,777	,802	1,248
HbA1c (%)	-6,257	1,842	-,293	-3,397	,001	-9,900	-2,614	,860	1,162
History of urinary tract infection=No	10,288	7,116	,118	1,446	,151	-3,788	24,364	,956	1,046
Chronic disease=No	6,507	8,496	,074	,766	,445	-10,298	23,312	,689	1,452

Note: F=3,529, $p = 0.002$; Adj. R^2=0.113. Variables included in the regression model: age, menopausal status (reference category: yes), duration of diabetes, body mass index, HbA1c, history of urinary tract infection (reference category: yes), presence of chronic disease (reference category: yes).

Model 2

Variables	Unstandardized coefficient		Standardized coefficient	t	p	95% CI		Collinearity statistics	
	B	SE	Beta			Lower	Upper	Tolerance	VIF
(Constant)	69,299	31,778		2,181	,031	6,433	132,164		
Age (years)	,442	,415	,137	1,065	,289	-,379	1,262	,339	2,954
Menopause=No	8,799	10,159	,103	,866	,388	-11,298	28,896	,399	2,504
Duration of diabetes (years)	-,389	,463	-,072	-,839	,403	-1,306	,528	,765	1,307
Body mass index (kg/m²)	,114	,448	,022	,254	,800	-,773	1,001	,786	1,272
HbA1c (%)	-2,718	1,909	-,127	-1,424	,157	-6,495	1,058	,705	1,419
History of urinary tract infection=No	7,316	6,710	,084	1,090	,278	-5,959	20,590	,946	1,057
Chronic disease=No	5,747	7,972	,065	,721	,472	-10,023	21,518	,688	1,453

BFLUTS-SF total score	-1,121	,257	-,392	-4,357	,000	-1,630	-,612	,693	1,442

Note: F=5,882, $p < 0.001$; Adj. R^2=0.219; ΔR^2=0.107, F change=18,986, $p < 0.001$. Variables included in the regression model: age, menopausal status (reference category: yes), duration of diabetes, body mass index, HbA1c, history of urinary tract infection (reference category: yes), presence of chronic disease (reference category: yes), BFLUTS-SF total score.

Vitality

Model 1

Variables	Unstandardized coefficient		Standardized coefficient	t	p	95% CI		Collinearity statistics	
	B	SE	Beta			Lower	Upper	Tolerance	VIF
(Constant)	70,707	11,741		6,022	,000	47,482	93,931		
Age (years)	-,024	,153	-,022	-,157	,876	-,326	,278	,341	2,932
Menopause=No	-3,356	3,757	-,114	-,893	,373	-10,787	4,076	,399	2,504
Duration of diabetes (years)	-,519	,171	-,279	-3,032	,003	-,858	-,181	,767	1,305
Body mass index (kg/m²)	-,041	,164	-,023	-,250	,803	-,366	,284	,802	1,248
HbA1c (%)	-1,271	,639	-,173	-1,989	,049	-2,535	-,007	,860	1,162
History of urinary tract infection=No	,504	2,469	,017	,204	,839	-4,380	5,387	,956	1,046
Chronic disease=No	3,114	2,947	,103	1,056	,293	-2,716	8,944	,689	1,452

Note: F=3,147, $p = 0.004$; Adj. R^2=0.098. Variables included in the regression model: age, menopausal status (reference category: yes), duration of diabetes, body mass index, HbA1c, history of urinary tract infection (reference category: yes), presence of chronic disease (reference category: yes).

Model 2

Variables	Unstandardized coefficient		Standardized coefficient	t	p	95% CI		Collinearity statistics	
	B	SE	Beta			Lower	Upper	Tolerance	VIF
(Constant)	68,293	10,768		6,342	,000	46,991	89,594		
Age (years)	,037	,141	,034	,266	,791	-,241	,315	,339	2,954
Menopause=No	-3,226	3,442	-,109	-,937	,350	-10,036	3,584	,399	2,504
Duration of diabetes (years)	-,488	,157	-,262	-3,106	,002	-,798	-,177	,765	1,307
Body mass index (kg/m²)	,067	,152	,037	,444	,658	-,233	,368	,786	1,272
HbA1c (%)	,139	,647	,019	,214	,831	-1,141	1,418	,705	1,419
History of urinary tract infection=No	-,680	2,274	-,023	-,299	,765	-5,178	3,818	,946	1,057
Chronic disease=No	2,811	2,701	,093	1,041	,300	-2,532	8,155	,688	1,453

BFLUTS-SF total score	-,447	,087	-,454	-5,122	,000	-,619	-,274	,693	1,442

Note: F=6,559, $p < 0.001$; Adj. R^2=0.242; ΔR^2=0.143, F change=26,233, $p < 0.001$. Variables included in the regression model: age, menopausal status (reference category: yes), duration of diabetes, body mass index, HbA1c, history of urinary tract infection (reference category: yes), presence of chronic disease (reference category: yes), BFLUTS-SF total score.

Mental Health

Model 1

**Variables**	**Unstandardized coefficient**		**Standardized coefficient**	**t**	**p**	**95% CI**		**Collinearity statistics**	
	**B**	**SE**	**Beta**			**Lower**	**Upper**	**Tolerance**	**VIF**
(Constant)	62,291	11,373		5,477	,000	39,793	84,788		
Age (years)	,118	,148	,109	,797	,427	-,175	,411	,341	2,932
Menopause=No	1,190	3,639	,041	,327	,744	-6,009	8,389	,399	2,504
Duration of diabetes (years)	-,662	,166	-,365	-3,991	,000	-,990	-,334	,767	1,305
Body mass index (kg/m²)	-,051	,159	-,029	-,321	,749	-,366	,264	,802	1,248
HbA1c (%)	-,742	,619	-,103	-1,198	,233	-1,966	,483	,860	1,162
History of urinary tract infection=No	-,963	2,391	-,033	-,403	,688	-5,693	3,767	,956	1,046
Chronic disease=No	2,000	2,855	,068	,700	,485	-3,648	7,648	,689	1,452

Note: F=3,405, p = 0.002; Adj. R^2=0.108. Variables included in the regression model: age, menopausal status (reference category: yes), duration of diabetes, body mass index, HbA1c, history of urinary tract infection (reference category: yes), presence of chronic disease (reference category: yes).

Model 2

**Variables**	**Unstandardized coefficient**		**Standardized coefficient**	**t**	**p**	**95% CI**		**Collinearity statistics**	
	**B**	**SE**	**Beta**			**Lower**	**Upper**	**Tolerance**	**VIF**
(Constant)	59,980	10,455		5,737	,000	39,297	80,663		
Age (years)	,177	,136	,164	1,295	,198	-,093	,447	,339	2,954
Menopause=No	1,315	3,342	,046	,393	,695	-5,298	7,927	,399	2,504
Duration of diabetes (years)	-,632	,152	-,348	-4,144	,000	-,934	-,330	,765	1,307
Body mass index (kg/m²)	,053	,147	,030	,359	,720	-,239	,345	,786	1,272
HbA1c (%)	,607	,628	,085	,967	,335	-,635	1,850	,705	1,419
History of urinary tract infection=No	-2,096	2,208	-,072	-,949	,344	-6,464	2,271	,946	1,057
Chronic disease=No	1,710	2,623	,058	,652	,516	-3,478	6,899	,688	1,453

BFLUTS-SF total score	-,427	,085	-,446	-5,049	,000	-,595	-,260	,693	1,442

Note: F=6,720, $p < 0.001$; Adj. R^2=0.248; ΔR^2=0.138, F change=25,495, $p < 0.001$. Variables included in the regression model: age, menopausal status (reference category: yes), duration of diabetes, body mass index, HbA1c, history of urinary tract infection (reference category: yes), presence of chronic disease (reference category: yes), BFLUTS-SF total score.

Social Functioning

Model 1

Variables	Unstandardized coefficient		Standardized coefficient	t	p	95% CI		Collinearity statistics	
	B	SE	Beta			Lower	Upper	Tolerance	VIF
(Constant)	88,833	17,888		4,966	,000	53,448	124,218		
Age (years)	,181	,233	,103	,779	,437	-,279	,642	,341	2,932
Menopause=No	4,715	5,724	,101	,824	,412	-6,607	16,038	,399	2,504
Duration of diabetes (years)	-,456	,261	-,155	-1,747	,083	-,972	,060	,767	1,305
Body mass index (kg/m²)	-,503	,250	-,174	-2,010	,046	-,997	-,008	,802	1,248
HbA1c (%)	-3,672	,973	-,315	-3,772	,000	-5,598	-1,746	,860	1,162
History of urinary tract infection=No	1,257	3,761	,026	,334	,739	-6,183	8,697	,956	1,046
Chronic disease=No	1,144	4,491	,024	,255	,799	-7,739	10,027	,689	1,452

Note: F=4,951, $p < 0.001$; Adj. R^2=0.166. Variables included in the regression model: age, menopausal status (reference category: yes), duration of diabetes, body mass index, HbA1c, history of urinary tract infection (reference category: yes), presence of chronic disease (reference category: yes).

Model 2

Variables	Unstandardized coefficient		Standardized coefficient	t	p	95% CI		Collinearity statistics	
	B	SE	Beta			Lower	Upper	Tolerance	VIF
(Constant)	83,218	14,051		5,923	,000	55,421	111,014		
Age (years)	,324	,183	,185	1,767	,080	-,039	,687	,339	2,954
Menopause=No	5,017	4,492	,107	1,117	,266	-3,869	13,903	,399	2,504
Duration of diabetes (years)	-,382	,205	-,130	-1,866	,064	-,788	,023	,765	1,307
Body mass index (kg/m²)	-,250	,198	-,087	-1,262	,209	-,642	,142	,786	1,272
HbA1c (%)	-,394	,844	-,034	-,467	,641	-2,064	1,276	,705	1,419
History of urinary tract infection=No	-1,497	2,967	-,032	-,504	,615	-7,366	4,373	,946	1,057
Chronic disease=No	,440	3,525	,009	,125	,901	-6,533	7,413	,688	1,453

https://doi.org/10.3390/healthcare14213753

BFLUTS-SF total score	-1,039	,114	-,667	-9,130	,000	-1,264	-,814	,693	1,442

Note: F=17,454, $p < 0.001$; Adj. R^2=0.486; ΔR^2=0.308, F change=83,352, $p < 0.001$. Variables included in the regression model: age, menopausal status (reference category: yes), duration of diabetes, body mass index, HbA1c, history of urinary tract infection (reference category: yes), presence of chronic disease (reference category: yes), BFLUTS-SF total score.

Bodily Pain

Model 1

**Variables**	**Unstandardized coefficient**		**Standardized coefficient**	**t**	**p**	**95% CI**		**Collinearity statistics**	
	**B**	**SE**	**Beta**			**Lower**	**Upper**	**Tolerance**	**VIF**
(Constant)	107,691	21,977		4,900	,000	64,218	151,164		
Age (years)	,362	,286	,161	1,267	,207	-,203	,928	,341	2,932
Menopause=No	5,324	7,032	,089	,757	,450	-8,586	19,235	,399	2,504
Duration of diabetes (years)	-,660	,321	-,175	-2,059	,041	-1,294	-,026	,767	1,305
Body mass index (kg/m²)	-,659	,307	-,178	-2,144	,034	-1,267	-,051	,802	1,248
HbA1c (%)	-6,155	1,196	-,412	-5,146	,000	-8,521	-3,789	,860	1,162
History of urinary tract infection=No	-1,468	4,621	-,024	-,318	,751	-10,609	7,672	,956	1,046
Chronic disease=No	1,769	5,517	,029	,321	,749	-9,144	12,682	,689	1,452

Note: F=7,058, $p < 0.001$; Adj. R^2=0.234. Variables included in the regression model: age, menopausal status (reference category: yes), duration of diabetes, body mass index, HbA1c, history of urinary tract infection (reference category: yes), presence of chronic disease (reference category: yes).

Model 2

**Variables**	**Unstandardized coefficient**		**Standardized coefficient**	**t**	**p**	**95% CI**		**Collinearity statistics**	
	**B**	**SE**	**Beta**			**Lower**	**Upper**	**Tolerance**	**VIF**
(Constant)	100,791	17,261		5,839	,000	66,645	134,937		
Age (years)	,538	,225	,239	2,387	,018	,092	,983	,339	2,954
Menopause=No	5,696	5,518	,095	1,032	,304	-5,220	16,612	,399	2,504
Duration of diabetes (years)	-,570	,252	-,151	-2,264	,025	-1,068	-,072	,765	1,307
Body mass index (kg/m²)	-,348	,243	-,094	-1,431	,155	-,830	,133	,786	1,272
HbA1c (%)	-2,127	1,037	-,142	-2,051	,042	-4,178	-,076	,705	1,419
History of urinary tract infection=No	-4,852	3,645	-,080	-1,331	,185	-12,062	2,358	,946	1,057
Chronic disease=No	,904	4,330	,015	,209	,835	-7,662	9,470	,688	1,453

BFLUTS-SF total score	-1,276	,140	-,639	-9,132	,000	-1,553	-1,000	,693	1,442

Note: F=20,455, p < 0.001; Adj. R^2=0.528; ΔR^2=0.283, F change=83,399, p < 0.001. Variables included in the regression model: age, menopausal status (reference category: yes), duration of diabetes, body mass index, HbA1c, history of urinary tract infection (reference category: yes), presence of chronic disease (reference category: yes), BFLUTS-SF total score.

General Health

Model 1

Variables	Unstandardized coefficient		Standardized coefficient	t	p	95% CI		Collinearity statistics	
	B	SE	Beta			Lower	Upper	Tolerance	VIF
(Constant)	50,462	13,358		3,778	,000	24,039	76,884		
Age (years)	,154	,174	,129	,886	,377	-,190	,498	,341	2,932
Menopause=No	4,356	4,274	,137	1,019	,310	-4,099	12,811	,399	2,504
Duration of diabetes (years)	-,103	,195	-,051	-,528	,599	-,488	,283	,767	1,305
Body mass index (kg/m²)	-,001	,187	-,001	-,007	,994	-,371	,368	,802	1,248
HbA1c (%)	-1,184	,727	-,149	-1,629	,106	-2,622	,254	,860	1,162
History of urinary tract infection=No	3,098	2,809	,096	1,103	,272	-2,458	8,653	,956	1,046
Chronic disease=No	,191	3,353	,006	,057	,955	-6,442	6,824	,689	1,452

Note: F=,948, p = 0.472; Adj. R^2=-.003. Variables included in the regression model: age, menopausal status (reference category: yes), duration of diabetes, body mass index, HbA1c, history of urinary tract infection (reference category: yes), presence of chronic disease (reference category: yes).

Model 2

Variables	Unstandardized coefficient		Standardized coefficient	t	p	95% CI		Collinearity statistics	
	B	SE	Beta			Lower	Upper	Tolerance	VIF
(Constant)	48,801	13,005		3,752	,000	23,073	74,529		
Age (years)	,196	,170	,164	1,156	,250	-,140	,532	,339	2,954
Menopause=No	4,445	4,158	,140	1,069	,287	-3,779	12,670	,399	2,504
Duration of diabetes (years)	-,081	,190	-,040	-,428	,670	-,456	,294	,765	1,307
Body mass index (kg/m²)	,073	,183	,037	,400	,690	-,290	,436	,786	1,272
HbA1c (%)	-,215	,781	-,027	-,275	,784	-1,760	1,331	,705	1,419
History of urinary tract infection=No	2,283	2,746	,071	,831	,407	-3,149	7,716	,946	1,057
Chronic disease=No	-,017	3,263	-,001	-,005	,996	-6,471	6,437	,688	1,453

BFLUTS-SF total score	-,307	,105	-,289	-2,917	,004	-,516	-,099	,693	1,442

Note: F=1,941, p = 0.059; Adj. R^2=0.051; ΔR^2=0.058, F change=8,511, p = 0.004. Variables included in the regression model: age, menopausal status (reference category: yes), duration of diabetes, body mass index, HbA1c, history of urinary tract infection (reference category: yes), presence of chronic disease (reference category: yes), BFLUTS-SF total score.

Supplementary Table S2. Sensitivity analysis of the association between the BFLUTS-LUTS score and SF-36 subscales using hierarchical multiple linear regression.

Physical Functioning

Model 1

Variables	Unstandardized coefficient		Standardized coefficient	t	p	95% CI		Collinearity statistics	
	B	SE	Beta			Lower	Upper	Tolerance	VIF
(Constant)	105,662	28,916		3,654	,000	48,463	162,862		
Age (years)	,209	,376	,073	,555	,580	-,536	,953	,341	2,932
Menopause=No	10,239	9,253	,134	1,107	,270	-8,064	28,542	,399	2,504
Duration of diabetes (years)	-,839	,422	-,174	-1,989	,049	-1,673	-,004	,767	1,305
Body mass index (kg/m²)	-,393	,404	-,083	-,972	,333	-1,193	,407	,802	1,248
HbA1c (%)	-6,339	1,574	-,333	-4,028	,000	-9,451	-3,226	,860	1,162
History of urinary tract infection=No	4,093	6,080	,053	,673	,502	-7,934	16,120	,956	1,046
Chronic disease=No	1,307	7,259	,017	,180	,857	-13,052	15,666	,689	1,452

Note: F=5,463, $p < 0.001$; Adj. R^2=0.184. Variables included in the regression model: age, menopausal status (reference category: yes), duration of diabetes, body mass index, HbA1c, history of urinary tract infection (reference category: yes), presence of chronic disease (reference category: yes).

Model 2

Variables	Unstandardized coefficient		Standardized coefficient	t	p	95% CI		Collinearity statistics	
	B	SE	Beta			Lower	Upper	Tolerance	VIF
(Constant)	93,476	25,752		3,630	,000	42,532	144,421		
Age (years)	,408	,336	,142	1,216	,226	-,256	1,073	,338	2,961
Menopause=No	11,077	8,216	,145	1,348	,180	-5,176	27,330	,399	2,504
Duration of diabetes (years)	-,660	,376	-,137	-1,757	,081	-1,403	,083	,762	1,313
Body mass index (kg/m²)	-,050	,363	-,011	-,137	,891	-,769	,669	,782	1,279
HbA1c (%)	-2,460	1,538	-,129	-1,600	,112	-5,502	,582	,710	1,408
History of urinary tract infection=No	2,063	5,409	,027	,381	,704	-8,637	12,762	,953	1,050

Chronic disease=No	,871	6,445	,011	,135	,893	-11,879	13,622	,688	1,453
BFLUTS-LUTS score	-1,870	,310	-,495	-6,038	,000	-2,483	-1,258	,689	1,451

Note: F=10,621, p < 0.001; Adj. R^2=0.356; ΔR^2=0.169, F change=36,458, p < 0.001. Variables included in the regression model: age, menopausal status (reference category: yes), duration of diabetes, body mass index, HbA1c, history of urinary tract infection (reference category: yes), presence of chronic disease (reference category: yes), BFLUTS-LUTS score (combined storage, voiding, and incontinence symptom score).

Role Physical

Model 1

**Variables**	**Unstandardized coefficient**		**Standardized coefficient**	**t**	**p**	**95% CI**		**Collinearity statistics**	
	**B**	**SE**	**Beta**			**Lower**	**Upper**	**Tolerance**	**VIF**
(Constant)	62,703	34,110		1,838	,068	-4,770	130,176		
Age (years)	,320	,444	,100	,721	,472	-,558	1,198	,341	2,932
Menopause=No	10,411	10,915	,122	,954	,342	-11,179	32,001	,399	2,504
Duration of diabetes (years)	-,311	,498	-,058	-,625	,533	-1,295	,673	,767	1,305
Body mass index (kg/m²)	-,075	,477	-,014	-,158	,875	-1,019	,868	,802	1,248
HbA1c (%)	-5,878	1,856	-,276	-3,167	,002	-9,550	-2,207	,860	1,162
History of urinary tract infection=No	8,646	7,172	,100	1,205	,230	-5,541	22,833	,956	1,046
Chronic disease=No	7,653	8,563	,087	,894	,373	-9,286	24,591	,689	1,452

Note: F=3,013, p = 0.006; Adj. R^2=0.092. Variables included in the regression model: age, menopausal status (reference category: yes), duration of diabetes, body mass index, HbA1c, history of urinary tract infection (reference category: yes), presence of chronic disease (reference category: yes).

Model 2

**Variables**	**Unstandardized coefficient**		**Standardized coefficient**	**t**	**p**	**95% CI**		**Collinearity statistics**	
	**B**	**SE**	**Beta**			**Lower**	**Upper**	**Tolerance**	**VIF**
(Constant)	50,329	31,453		1,600	,112	-11,893	112,550		
Age (years)	,523	,410	,163	1,275	,205	-,288	1,334	,338	2,961
Menopause=No	11,262	10,035	,132	1,122	,264	-8,589	31,114	,399	2,504
Duration of diabetes (years)	-,129	,459	-,024	-,282	,778	-1,037	,778	,762	1,313
Body mass index (kg/m²)	,273	,444	,052	,615	,540	-,605	1,151	,782	1,279
HbA1c (%)	-1,940	1,878	-,091	-1,033	,304	-5,655	1,776	,710	1,408
History of urinary tract infection=No	6,584	6,606	,076	,997	,321	-6,484	19,652	,953	1,050

Chronic disease=No	7,210	7,872	,082	,916	,361	-8,363	22,783	,688	1,453
BFLUTS-LUTS score	-1,899	,378	-,449	-5,020	,000	-2,648	-1,151	,689	1,451

Note: F=6,269, $p < 0.001$; Adj. R^2=0.233; ΔR^2=0.139, F change=25,201, $p < 0.001$. Variables included in the regression model: age, menopausal status (reference category: yes), duration of diabetes, body mass index, HbA1c, history of urinary tract infection (reference category: yes), presence of chronic disease (reference category: yes), BFLUTS-LUTS score (combined storage, voiding, and incontinence symptom score).

Role Emotional

Model 1

Variables	Unstandardized coefficient		Standardized coefficient	t	p	95% CI		Collinearity statistics	
	B	SE	Beta			Lower	Upper	Tolerance	VIF
(Constant)	75,359	33,842		2,227	,028	8,417	142,302		
Age (years)	,288	,440	,089	,653	,515	-,583	1,159	,341	2,932
Menopause=No	8,472	10,829	,099	,782	,435	-12,948	29,893	,399	2,504
Duration of diabetes (years)	-,468	,494	-,087	-,948	,345	-1,445	,508	,767	1,305
Body mass index (kg/m²)	-,159	,473	-,030	-,335	,738	-1,095	,777	,802	1,248
HbA1c (%)	-6,257	1,842	-,293	-3,397	,001	-9,900	-2,614	,860	1,162
History of urinary tract infection=No	10,288	7,116	,118	1,446	,151	-3,788	24,364	,956	1,046
Chronic disease=No	6,507	8,496	,074	,766	,445	-10,298	23,312	,689	1,452

Note: F=3,529, p = 0.002; Adj. R^2=0.113. Variables included in the regression model: age, menopausal status (reference category: yes), duration of diabetes, body mass index, HbA1c, history of urinary tract infection (reference category: yes), presence of chronic disease (reference category: yes).

Model 2

Variables	Unstandardized coefficient		Standardized coefficient	t	p	95% CI		Collinearity statistics	
	B	SE	Beta			Lower	Upper	Tolerance	VIF
(Constant)	64,359	31,792		2,024	,045	1,466	127,252		
Age (years)	,468	,415	,145	1,129	,261	-,352	1,288	,338	2,961
Menopause=No	9,229	10,143	,108	,910	,365	-10,837	29,294	,399	2,504
Duration of diabetes (years)	-,307	,464	-,057	-,661	,510	-1,224	,611	,762	1,313
Body mass index (kg/m²)	,151	,449	,028	,336	,737	-,737	1,038	,782	1,279
HbA1c (%)	-2,755	1,898	-,129	-1,451	,149	-6,511	1,000	,710	1,408
History of urinary tract infection=No	8,455	6,677	,097	1,266	,208	-4,754	21,664	,953	1,050

Variables	B	SE	Beta	t	p	Lower	Upper	Tolerance	VIF
Chronic disease=No	6,114	7,957	,069	,768	,444	-9,627	21,855	,688	1,453
BFLUTS-LUTS score	-1,688	,382	-,398	-4,415	,000	-2,445	-,932	,689	1,451

Note: F=5,957, $p < 0.001$; Adj. R^2=0.222; ΔR^2=0.109, F change=19,493, $p < 0.001$. Variables included in the regression model: age, menopausal status (reference category: yes), duration of diabetes, body mass index, HbA1c, history of urinary tract infection (reference category: yes), presence of chronic disease (reference category: yes), BFLUTS-LUTS score (combined storage, voiding, and incontinence symptom score).

Vitality

Model 1

Variables	Unstandardized coefficient		Standardized coefficient	t	p	95% CI		Collinearity statistics	
	B	SE	Beta			Lower	Upper	Tolerance	VIF
(Constant)	70,707	11,741		6,022	,000	47,482	93,931		
Age (years)	-,024	,153	-,022	-,157	,876	-,326	,278	,341	2,932
Menopause=No	-3,356	3,757	-,114	-,893	,373	-10,787	4,076	,399	2,504
Duration of diabetes (years)	-,519	,171	-,279	-3,032	,003	-,858	-,181	,767	1,305
Body mass index (kg/m²)	-,041	,164	-,023	-,250	,803	-,366	,284	,802	1,248
HbA1c (%)	-1,271	,639	-,173	-1,989	,049	-2,535	-,007	,860	1,162
History of urinary tract infection=No	,504	2,469	,017	,204	,839	-4,380	5,387	,956	1,046
Chronic disease=No	3,114	2,947	,103	1,056	,293	-2,716	8,944	,689	1,452

Note: F=3,147, p = 0.004; Adj. R^2=0.098. Variables included in the regression model: age, menopausal status (reference category: yes), duration of diabetes, body mass index, HbA1c, history of urinary tract infection (reference category: yes), presence of chronic disease (reference category: yes).

Model 2

Variables	Unstandardized coefficient		Standardized coefficient	t	p	95% CI		Collinearity statistics	
	B	SE	Beta			Lower	Upper	Tolerance	VIF
(Constant)	66,055	10,624		6,218	,000	45,038	87,072		
Age (years)	,052	,139	,047	,377	,706	-,222	,326	,338	2,961
Menopause=No	-3,036	3,389	-,103	-,896	,372	-9,741	3,669	,399	2,504
Duration of diabetes (years)	-,451	,155	-,242	-2,910	,004	-,758	-,144	,762	1,313
Body mass index (kg/m²)	,090	,150	,049	,599	,550	-,207	,386	,782	1,279
HbA1c (%)	,210	,634	,029	,331	,741	-1,045	1,465	,710	1,408
History of urinary tract infection=No	-,271	2,231	-,009	-,122	,903	-4,685	4,143	,953	1,050

Chronic disease=No	2,948	2,659	,097	1,109	,270	-2,312	8,208	,688	1,453
BFLUTS-LUTS score	-,714	,128	-,489	-5,587	,000	-,967	-,461	,689	1,451

Note: F=7,285, $p < 0.001$; Adj. R^2=0.266; ΔR^2=0.165, F change=31,213, $p < 0.001$. Variables included in the regression model: age, menopausal status (reference category: yes), duration of diabetes, body mass index, HbA1c, history of urinary tract infection (reference category: yes), presence of chronic disease (reference category: yes), BFLUTS-LUTS score (combined storage, voiding, and incontinence symptom score).

Mental Health

Model 1

Variables	Unstandardized coefficient		Standardized coefficient	t	p	95% CI		Collinearity statistics	
	B	SE	Beta			Lower	Upper	Tolerance	VIF
(Constant)	62,291	11,373		5,477	,000	39,793	84,788		
Age (years)	,118	,148	,109	,797	,427	-,175	,411	,341	2,932
Menopause=No	1,190	3,639	,041	,327	,744	-6,009	8,389	,399	2,504
Duration of diabetes (years)	-,662	,166	-,365	-3,991	,000	-,990	-,334	,767	1,305
Body mass index (kg/m²)	-,051	,159	-,029	-,321	,749	-,366	,264	,802	1,248
HbA1c (%)	-,742	,619	-,103	-1,198	,233	-1,966	,483	,860	1,162
History of urinary tract infection=No	-,963	2,391	-,033	-,403	,688	-5,693	3,767	,956	1,046
Chronic disease=No	2,000	2,855	,068	,700	,485	-3,648	7,648	,689	1,452

Note: F=3,405, $p = 0.002$; Adj. R^2=0.108. Variables included in the regression model: age, menopausal status (reference category: yes), duration of diabetes, body mass index, HbA1c, history of urinary tract infection (reference category: yes), presence of chronic disease (reference category: yes).

Model 2

Variables	Unstandardized coefficient		Standardized coefficient	t	p	95% CI		Collinearity statistics	
	B	SE	Beta			Lower	Upper	Tolerance	VIF
(Constant)	58,093	10,452		5,558	,000	37,416	78,770		
Age (years)	,187	,136	,173	1,371	,173	-,083	,456	,338	2,961
Menopause=No	1,479	3,335	,052	,443	,658	-5,118	8,076	,399	2,504
Duration of diabetes (years)	-,601	,152	-,331	-3,939	,000	-,902	-,299	,762	1,313
Body mass index (kg/m²)	,067	,148	,038	,455	,650	-,225	,359	,782	1,279
HbA1c (%)	,594	,624	,083	,952	,343	-,640	1,829	,710	1,408
History of urinary tract infection=No	-1,662	2,195	-,057	-,757	,450	-6,005	2,680	,953	1,050

Chronic disease=No	1,850	2,616	,063	,707	,481	-3,325	7,025	,688	1,453
BFLUTS-LUTS score	-,644	,126	-,453	-5,124	,000	-,893	-,396	,689	1,451

Note: F=6,832, $p < 0.001$; Adj. R^2=0.251; ΔR^2=0.141, F change=26,259, $p < 0.001$. Variables included in the regression model: age, menopausal status (reference category: yes), duration of diabetes, body mass index, HbA1c, history of urinary tract infection (reference category: yes), presence of chronic disease (reference category: yes), BFLUTS-LUTS score (combined storage, voiding, and incontinence symptom score).

Social Functioning

Model 1

**Variables**	**Unstandardized coefficient**		**Standardized coefficient**	**t**	**p**	**95% CI**		**Collinearity statistics**	
	**B**	**SE**	**Beta**			**Lower**	**Upper**	**Tolerance**	**VIF**
(Constant)	88,833	17,888		4,966	,000	53,448	124,218		
Age (years)	,181	,233	,103	,779	,437	-,279	,642	,341	2,932
Menopause=No	4,715	5,724	,101	,824	,412	-6,607	16,038	,399	2,504
Duration of diabetes (years)	-,456	,261	-,155	-1,747	,083	-,972	,060	,767	1,305
Body mass index (kg/m²)	-,503	,250	-,174	-2,010	,046	-,997	-,008	,802	1,248
HbA1c (%)	-3,672	,973	-,315	-3,772	,000	-5,598	-1,746	,860	1,162
History of urinary tract infection=No	1,257	3,761	,026	,334	,739	-6,183	8,697	,956	1,046
Chronic disease=No	1,144	4,491	,024	,255	,799	-7,739	10,027	,689	1,452

Note: F=4,951, $p < 0.001$; Adj. R^2=0.166. Variables included in the regression model: age, menopausal status (reference category: yes), duration of diabetes, body mass index, HbA1c, history of urinary tract infection (reference category: yes), presence of chronic disease (reference category: yes).

Model 2

**Variables**	**Unstandardized coefficient**		**Standardized coefficient**	**t**	**p**	**95% CI**		**Collinearity statistics**	
	**B**	**SE**	**Beta**			**Lower**	**Upper**	**Tolerance**	**VIF**
(Constant)	78,653	13,986		5,624	,000	50,984	106,321		
Age (years)	,348	,182	,198	1,910	,058	-,013	,709	,338	2,961
Menopause=No	5,415	4,462	,116	1,214	,227	-3,412	14,242	,399	2,504
Duration of diabetes (years)	-,306	,204	-,104	-1,502	,136	-,710	,097	,762	1,313
Body mass index (kg/m²)	-,216	,197	-,075	-1,096	,275	-,607	,174	,782	1,279
HbA1c (%)	-,432	,835	-,037	-,517	,606	-2,084	1,220	,710	1,408
History of urinary tract infection=No	-,439	2,937	-,009	-,150	,881	-6,250	5,372	,953	1,050

Chronic disease=No	,780	3,501	,016	,223	,824	-6,145	7,705	,688	1,453
BFLUTS-LUTS score	-1,562	,168	-,676	-9,287	,000	-1,895	-1,230	,689	1,451

Note: F=17,913, $p < 0.001$; Adj. R^2=0.493; ΔR^2=0.314, F change=86,257, $p < 0.001$. Variables included in the regression model: age, menopausal status (reference category: yes), duration of diabetes, body mass index, HbA1c, history of urinary tract infection (reference category: yes), presence of chronic disease (reference category: yes), BFLUTS-LUTS score (combined storage, voiding, and incontinence symptom score).

Bodily Pain

Model 1

Variables	Unstandardized coefficient		Standardized coefficient	t	p	95% CI		Collinearity statistics	
	B	SE	Beta			Lower	Upper	Tolerance	VIF
(Constant)	107,691	21,977		4,900	,000	64,218	151,164		
Age (years)	,362	,286	,161	1,267	,207	-,203	,928	,341	2,932
Menopause=No	5,324	7,032	,089	,757	,450	-8,586	19,235	,399	2,504
Duration of diabetes (years)	-,660	,321	-,175	-2,059	,041	-1,294	-,026	,767	1,305
Body mass index (kg/m²)	-,659	,307	-,178	-2,144	,034	-1,267	-,051	,802	1,248
HbA1c (%)	-6,155	1,196	-,412	-5,146	,000	-8,521	-3,789	,860	1,162
History of urinary tract infection=No	-1,468	4,621	-,024	-,318	,751	-10,609	7,672	,956	1,046
Chronic disease=No	1,769	5,517	,029	,321	,749	-9,144	12,682	,689	1,452

Note: F=7,058, $p < 0.001$; Adj. R^2=0.234. Variables included in the regression model: age, menopausal status (reference category: yes), duration of diabetes, body mass index, HbA1c, history of urinary tract infection (reference category: yes), presence of chronic disease (reference category: yes).

Model 2

Variables	Unstandardized coefficient		Standardized coefficient	t	p	95% CI		Collinearity statistics	
	B	SE	Beta			Lower	Upper	Tolerance	VIF
(Constant)	95,410	17,385		5,488	,000	61,019	129,801		
Age (years)	,564	,227	,251	2,487	,014	,115	1,012	,338	2,961
Menopause=No	6,169	5,546	,103	1,112	,268	-4,803	17,141	,399	2,504
Duration of diabetes (years)	-,480	,254	-,127	-1,892	,061	-,982	,022	,762	1,313
Body mass index (kg/m²)	-,313	,245	-,085	-1,276	,204	-,798	,172	,782	1,279
HbA1c (%)	-2,246	1,038	-,150	-2,164	,032	-4,300	-,193	,710	1,408
History of urinary tract infection=No	-3,514	3,651	-,058	-,963	,338	-10,737	3,708	,953	1,050

Chronic disease=No	1,330	4,351	,022	,306	,760	-7,277	9,938	,688	1,453
BFLUTS-LUTS score	-1,885	,209	-,636	-9,014	,000	-2,299	-1,471	,689	1,451

Note: F=20,087, $p < 0.001$; Adj. R^2=0.523; ΔR^2=0.279, F change=81,255, $p < 0.001$. Variables included in the regression model: age, menopausal status (reference category: yes), duration of diabetes, body mass index, HbA1c, history of urinary tract infection (reference category: yes), presence of chronic disease (reference category: yes), BFLUTS-LUTS score (combined storage, voiding, and incontinence symptom score).

General Health

Model 1

Variables	Unstandardized coefficient		Standardized coefficient	t	p	95% CI		Collinearity statistics	
	B	SE	Beta			Lower	Upper	Tolerance	VIF
(Constant)	50,462	13,358		3,778	,000	24,039	76,884		
Age (years)	,154	,174	,129	,886	,377	-,190	,498	,341	2,932
Menopause=No	4,356	4,274	,137	1,019	,310	-4,099	12,811	,399	2,504
Duration of diabetes (years)	-,103	,195	-,051	-,528	,599	-,488	,283	,767	1,305
Body mass index (kg/m²)	-,001	,187	-,001	-,007	,994	-,371	,368	,802	1,248
HbA1c (%)	-1,184	,727	-,149	-1,629	,106	-2,622	,254	,860	1,162
History of urinary tract infection=No	3,098	2,809	,096	1,103	,272	-2,458	8,653	,956	1,046
Chronic disease=No	,191	3,353	,006	,057	,955	-6,442	6,824	,689	1,452

Note: F=0,948, p = 0.472; Adj. R^2=-0.003. Variables included in the regression model: age, menopausal status (reference category: yes), duration of diabetes, body mass index, HbA1c, history of urinary tract infection (reference category: yes), presence of chronic disease (reference category: yes).

Model 2

Variables	Unstandardized coefficient		Standardized coefficient	t	p	95% CI		Collinearity statistics	
	B	SE	Beta			Lower	Upper	Tolerance	VIF
(Constant)	47,463	13,028		3,643	,000	21,691	73,235		
Age (years)	,203	,170	,170	1,196	,234	-,133	,539	,338	2,961
Menopause=No	4,562	4,156	,143	1,098	,274	-3,660	12,785	,399	2,504
Duration of diabetes (years)	-,059	,190	-,029	-,309	,757	-,435	,317	,762	1,313
Body mass index (kg/m²)	,083	,184	,042	,452	,652	-,281	,447	,782	1,279
HbA1c (%)	-,230	,778	-,029	-,295	,768	-1,769	1,309	,710	1,408
History of urinary tract infection=No	2,598	2,736	,080	,950	,344	-2,815	8,011	,953	1,050

Chronic disease=No	,084	3,261	,003	,026	,979	-6,366	6,534	,688	1,453
BFLUTS-LUTS score	-,460	,157	-,292	-2,937	,004	-,770	-,150	,689	1,451

Note: F=1,956, p = 0.057; Adj. R^2=0.052; ΔR^2=0.059, F change=8,628, p = 0.004. Variables included in the regression model: age, menopausal status (reference category: yes), duration of diabetes, body mass index, HbA1c, history of urinary tract infection (reference category: yes), presence of chronic disease (reference category: yes), BFLUTS-LUTS score (combined storage, voiding, and incontinence symptom score).

Disclaimer/Publisher's Note: The statements, opinions and data contained in all publications are solely those of the individual author(s) and contributor(s) and not of MDPI and/or the editor(s). MDPI and/or the editor(s) disclaim responsibility for any injury to people or property resulting from any ideas, methods, instructions or products referred to in the content.

https://doi.org/10.3390/healthcare14213753
